# In Situ Potentiometric Monitoring of Nitrate Removal from Aqueous Solution by Activated Carbon and Ion Exchange Resin

**DOI:** 10.3390/mi15111366

**Published:** 2024-11-12

**Authors:** José Manuel Olmos, Lucía Gil, Joaquín Ángel Ortuño

**Affiliations:** Department of Analytical Chemistry, University of Murcia, 30100 Murcia, Spain; luciagilso38@gmail.com

**Keywords:** ion-selective electrode, nitrate removal, activated carbon, ion exchange resin, dynamic potential response, kinetics characterization

## Abstract

A nitrate selective electrode was used for real-time in situ potentiometric monitoring of a batch nitrate removal process using activated carbon and ion exchange resin. A plasticized polymeric membrane consisting of polyvinyl chloride, 2-nitrophenyl octyl ether and tridodecyl methyl ammonium chloride was incorporated into an ion-selective electrode body. First, the dynamic potential response of the electrode to nitrate was investigated. Two commercial activated carbons with different physical properties were then tested. Nitrate removal with these carbons was monitored potentiometrically using several nitrate concentrations. The extreme turbidity of the solutions was not a drawback during potentiometric monitoring of the process, which is a clear advantage over other methods such as optical monitoring. The potential versus time recordings were converted into nitrate concentration versus time plots, which were evaluated with different adsorption kinetic models. A pseudo-second order kinetic model for nitrate adsorption on both activated carbons was found to fit the experimental data very well. The values of the kinetic parameters were very different between the two activated carbons. The proposed methodology was also satisfactorily applied to the study of nitrate removal by an ion exchange resin. In this case, the experimental results clearly follow a pseudo-first order kinetic model. Potential applications of the proposed methodology for monitoring nitrate removal in real water samples are discussed.

## 1. Introduction

Due to the high solubility of its salts in water, nitrate is a common pollutant in groundwater and surface water. The occurrence of nitrate in high concentrations is mainly caused by the intensive use of nitrogen-based fertilizers and pesticides in agriculture, as well as by uncontrolled domestic and industrial wastewater, livestock farming and septic tank overflows [1,2,3]. Consumption of water with high concentrations of nitrate has been linked to the development of diseases such as hypertension and methaemoglobinaemia, thyroid problems, cytogenetic defects, stomach cancer, birth defects, immune system alterations and respiratory infections [4,5]. As a result, the World Health Organization (WHO) has set a maximum limit of 50 mg/L of nitrate in drinking water [6]. Excessive nitrate in aquatic environments also causes rapid growth of plants and algae, which results in drastic oxygen depletion, leading to irreversible changes in the ecosystem and massive mortality of living organisms [7].

The potential toxicity of nitrate has motivated the development of experimental methodologies for its removal from water. These approaches can be divided into two groups. First, nitrate can be reduced to less hazardous compounds by chemical methods (photochemical, electrochemical or metallic reduction) or by biological methods (autotrophic and heterotrophic denitrification) [8,9,10,11,12]. Secondly, physical separation methods such as the use of membranes, ion exchange processes and adsorption to porous materials can be used [13,14,15,16,17]. Adsorption of nitrate is one of the main methods for its removal from solution due to its simplicity, ease of operation and low cost. Several materials can be employed, although the most used adsorbents are activated carbons and organic polymers such as chitosan and cellulose. Anion exchange resins are also commonly employed for the removal of nitrate, offering a simple methodology with a great operating capacity and a low treatmentcost.

The amount of nitrate in solution can be quantified by several methods, including UV–visible spectrophotometry, spectrofluorimetry, capillary electrophoresis, chromatographic techniques, (electro)chemiluminescent methods, voltammetry, coulometry and potentiometry with ion-selective electrodes [18,19,20]. However, the use of these methods to monitor nitrate removal from aqueous solution requires the extraction of sample aliquots at different times during the process and subsequent analytical determination of the analyte. In addition, the methods are further complicated when adsorption in batch solution is used for nitrate removal, since in this case a prior step involving separating the adsorbent from the aliquots is required for the determination of the residual nitrate concentration in the solution. As a result, the existing procedures are usually quite tedious and time consuming.

In this study, a nitrate selective electrode is used for in situ potentiometric monitoring of nitrate removal from a batch solution. To our knowledge, this approach has not been reported before and has advantages over other existing methods in that it allows real-time monitoring and avoids any separation step of the adsorbent prior to measurement. As a proof of concept, activated carbon and ion exchange resins have been used as materials for nitrate removal from aqueous solutions. This is challenging as the activated carbon suspensions used are extremely turbid and, therefore, do not allow optical monitoring in situ.

Several nitrate selective electrodes have been reported in the literature [21,22,23,24,25], but to our knowledge none have been used to monitor in situ nitrate removal by adsorbents. Although the main aim of this paper is to demonstrate the feasibility and advantages of in situ potentiometric monitoring of nitrate removal by different materials, the dynamic potentiometric response, the calibration curve equation and some aspects of the selectivity of the nitrate selective electrode constructed here have also been included.

## 2. Materials and Methods

### 2.1. Reagents and Solutions

High molecular weight poly(vinyl chloride) (PVC), 2-nitrophenyl octyl ether (NPOE), tridodecyl methyl ammonium chloride (TDMACl) and tetrahydrofuran (THF) of Selectophore grade were obtained from Sigma-Aldrich (Burlington, MA, USA). Two activated carbons—DARCO^®^ 100 mesh powder activated charcoal (activated carbon 1) and activated charcoal acid-washed with hydrochloric acid—(activated carbon 2) were obtainedfrom Sigma Aldrich. Anion exchange resin Ambersep^®^ 900 (hydroxide form, 16–45 mesh, strongly basic) was obtained from Merck (Darmstadt, Germany). All other reagents were of analytical grade. Milli-Q water obtained with a purification system acquired from Merck was used throughout.

### 2.2. Instrumentation

**Figure 1:**
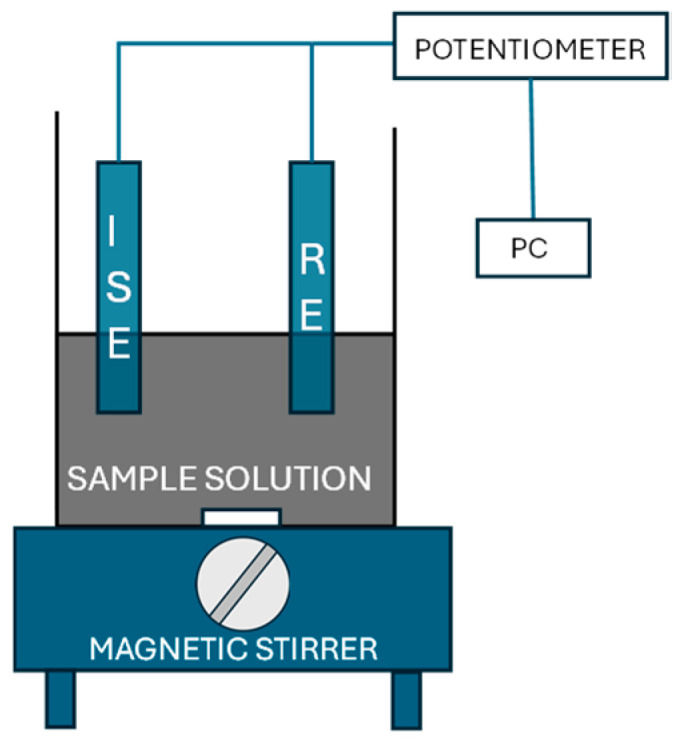
A scheme of the experimental setup employed for the potentiometric measurements. ISE, nitrate selective. RE, reference electrode.

A Fluka (Buchs, Switzerland) ISE body and a Thermo Scientific Orion Ag/AgCl double junction reference electrode (Orion 900200) acquired from Thermo Fisher Scientific (Waltham, MA, USA) with 0.1 M KCl solution in the outer compartment were used. A homemade potentiometer with high impedance data acquisition was connected to a personal computer via USB and software for potentiometric measurements was used. All potentiometric measurements were performed with constant stirring using an IKA (Staufen, Germany) Color Squid magnetic stirrer. A simplified scheme of the experimental setup is shown in Figure 1.

### 2.3. Construction of the Ion-Selective Electrode

The electrode membrane was prepared by dissolving 99.1 mg PVC, 200.24 mg NPOE and 2.79 mg TDMACl in 3 mL THF. The resulting solution was poured into a Fluka glass plate and allowed to stand for 24 h to ensure complete evaporation of the solvent. A circular section of the membrane was punched out and inserted into the ISE body. A solution of 1 × 10^−^^2^ M KCl and 1 × 10^−^^2^ M KNO_3_ M was used as both the internal solution and the storage solution for the ion-selective electrode.

The electrode was conditioned by immersing it in a 1 × 10^−^^3^ M KNO_3_ solution for several days to completely displace the chloride anion in the membrane by nitrate. The electrode was also stored in a 1 × 10^−^^3^ M KNO_3_ solution.

### 2.4. Calibration of the Electrode

The ISE and the reference electrode were immersed in 50 mL Milli-Q water with constant stirring until the potential stabilized. Appropriate aliquots of 0.01 and 1 M KNO_3_ standard solutions were then added to achieve different nitrate concentrations in the range of 1 × 10^−^^6^–1 × 10^−^^2^ M. After each aliquot addition, the potential was allowed to reach a stable value before a new addition was made. The potential response was recorded continuously at a data acquisition rate of one measurement per second. To construct the calibration graph, the stable potential values reached after each addition were plotted against the corresponding nitrate concentration obtained in the solution from the corresponding accumulated additions.

### 2.5. Pretreatment of the Anion Exchange Resin

The anion exchange resin was conditioned in Milli-Q water. Successive washes were performed until the resulting supernatant reached a pH of 7.5.

### 2.6. Potentiometric Monitoring of the Removal of Nitrate

The procedure for monitoring nitrate removal was identical for the two types of activated carbon and anion exchange resin used. First, the ISE and reference electrodes were immersed in 50 mL of Milli-Q water with constant stirring and allowed to approximately stabilize in potential. An aliquot of nitrate solution was then added to achieve the desired concentration in the solution. When the potential stabilized at the new value, 0.05 g of activated carbon or ion exchange resin was added to the sample solution and the adsorption process was maintained for the required time.

The dynamic potential vs. time record was transformed into the corresponding concentration vs. time plot by using a recent calibration plot covering the appropriate nitrate concentration of the adsorption process studied (see Section 2.4).

## 3. Results and Discussion

Anion-selective electrodes based on quaternary ammonium salts dissolved in a liquid or plasticized polymeric membrane respond to inorganic anions, according to the Hofmeister series [26,27]. This is due to the relative affinity of the anions between the aqueous and membrane phases and allows the relatively high selectivity of nitrate over other common anions, such as sulphate, mono- and dihydrogen phosphate, hydrogen carbonate and chloride, without the need to use a nitrate selective ionophore. In addition, the approach based on using an ionophore to improve selectivity is not as efficient for nitrate as for some other ions due to the lack of highly selective ionophores for nitrate [28].

### 3.1. Potentiometric Response of the Electrode to Nitrate in Aqueous Solution

**Figure 2:**
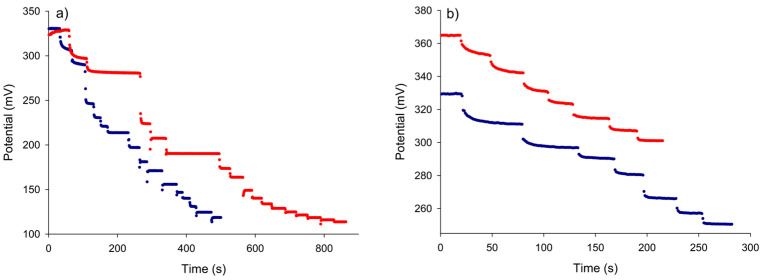
The dynamic potential response of the nitrate electrode over a wide concentration range (**a**) and a narrow concentration range (**b**). The blue curve in (**a**) corresponds to a calibration performed six weeks after the red one. The blue curve in (**b**) corresponds to a different membrane than that used in the red curve.

Prior to using the constructed nitrate selective electrode for in situ monitoring of the nitrate removal process in water, the analytical performance of the electrode in the quantification of nitrate in aqueous solution was investigated. Following the procedure described in Section 2.4, the dynamic potential response of the ISE was recorded for different nitrate concentrations. Figure 2a,b show the dynamic potential responses of the electrode for two different concentration ranges of nitrate, a wide range (5 × 10^−^^6^ M–9.3 × 10^−^^3^ M) and a narrow range at a low concentration (1 × 10^−^^6^ M–4 × 10^−^^5^ M), respectively. Results obtained on two different days separated by six weeks for the wide concentration range are shown in Figure 2a and those obtained for two different membranes are shown in Figure 2b. The electrode’s response to changes in nitrate concentration was fast in all cases, showing a sudden potential drop after each concentration increase before quickly reaching a new stable potential value. Note that in Figure 2a two concentrations were left for a longer period of time to confirm the absence of potential drift.

The corresponding calibration plots, constructed from the final potentials reached at each concentration versus the nitrate concentration obtained with the corresponding accumulated additions, were obtained. Excellent linearity was obtained for the calibrations corresponding to the wide concentration range. The experimental points were fitted to the Nernst Equation (1):

(1) $$E=E^{0’}+S\log C$$

where E is the measured potential, $E^{0´}$ is the formal potential, S is the slope of the calibration line and C is the molar concentration of nitrate in the solution. Optimum values of $E^{0´}$ and S were obtained from the fitting and they are summarized in Table 1. As can be seen, despite the long period elapsed between the two calibrations (6 weeks), the parameters are similar in both cases and the slope S is close to the Nernstian value of −59 mV/dec. The potential shift for 5 × 10^−^^6^ M was 9.6 mV over 6 weeks and the corresponding slope change was 0.9 mV/dec. As with all selective electrodes, periodic calibration is recommended prior to use.

In the case of calibration over a narrow range of low nitrate concentrations, the relationship between the potential and the nitrate concentration is not completely linear over the entire range, as the plot line curves smoothly at the lower concentrations. The following equation, which has already been reported [25] as including the part of the ion-selective electrode calibration curve at very low concentrations, was used to fit the experimental data:

(2) $$E=E^{0´}+S\log(C+LD)$$

where the *LD* constant corresponds to the detection limit of the ion-selective electrode according to the IUPAC criteria, as it corresponds to the concentration at the intersection of the extrapolated linear region and the last low concentration level segment of the calibration curve [29].

The values of the fitting parameters obtained for the calibrations of two different membranes in this concentration range are shown in Table 2. Both membranes showed a good potential response to low nitrate concentration, but the calibration parameters obtained were somewhat different between them, especially the $E^{0´}$ value. This can be attributed to the different times of use of both membranes at the time of calibration and can be resolved by performing a calibration prior to use.

### 3.2. Nitrate Removal by Activated Carbon Adsorption

#### 3.2.1. Potentiometric Monitoring of Nitrate Removal

**Figure 3:**
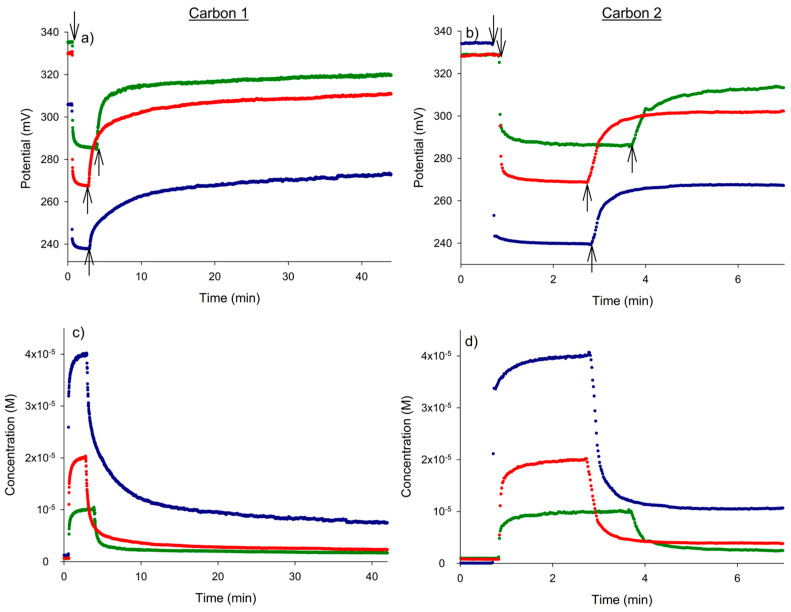
The dynamic potential responses (**a**,**b**) and corresponding nitrate concentration vs. time plots (**c**,**d**) obtained for the nitrate removal process with activated carbons 1 and 2 for three initial nitrate concentrations, 1 × 10^−5^ (green), 2 × 10^−5^ (red) and 4 × 10^−5^ M (blue). The first and second arrows in Figure 2a,b correspond to the addition of nitrate and activated carbon, respectively.

Once the electrode was correctly calibrated for the determination of nitrate in aqueous solution, it was used to monitor its removal by adsorption on two types of activated carbon. Figure 3a,b show the potential–time recordings corresponding to three different initial nitrate concentrations (1 × 10^−^^5^ M, 2 × 10^−^^5^ M and 4 × 10^−^^5^ M) subjected to activated carbons 1 and 2, respectively. The corresponding nitrate concentration versus time plots are shown in Figure 3c and Figure 3d, respectively.

The first sharp drop in potential that was observed corresponded to the injection of nitrate into the water. Thus, the initial potential of the water rapidly evolved to a new stable value depending on the nitrate concentration obtained in the solution through each addition. The addition of activated carbon then led to a continuous increase in potential, reflecting the continuous decrease in the nitrate’s concentration due to its adsorption on the activated carbon. The time evolution of the potential to a constant value was very different for the two carbons used. The new constant potential was reached after 4 min with the addition of activated carbon 2, whereas 40 min was required for activated carbon 1 to reach an almost constant value. Note that the final potential values reached did not match the corresponding initial potential values in pure water, indicating that the process of nitrate removal from the solution was not fully complete.

**Figure 4:**
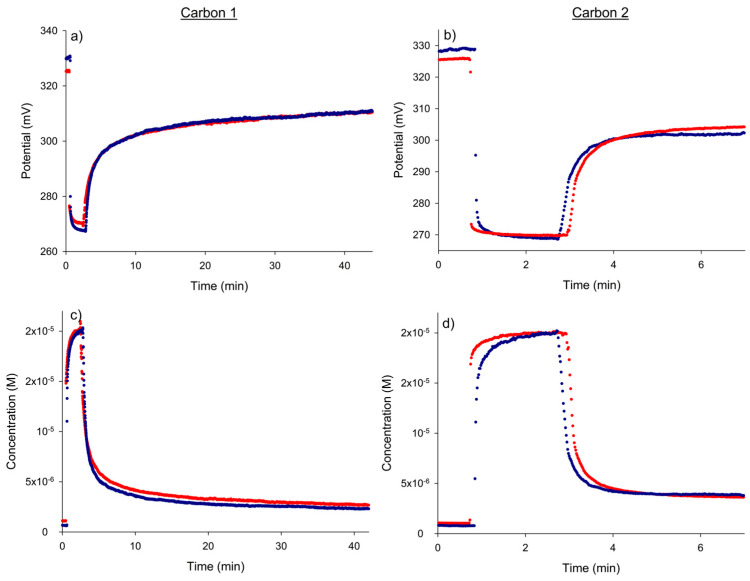
The between-day reproducibility of the dynamic potential responses (**a**,**b**) and the corresponding nitrate concentration vs. time plots (**c**,**d**) obtained for the nitrate removal process with activated carbons 1 and 2 for an initial nitrate concentration of 2 × 10^−5^ M.

The reproducibility of the adsorption process and the corresponding potentiometric monitoring was checked by repeating the adsorption experiment with 2 × 10^−^^5^ M nitrate on another day (Figure 4). The degree of agreement of the potential–time and concentration–time responses was excellent.

The results shown in Figure 3c,d and Figure 4 allow us to determine the effectiveness of the nitrate removal process with both types of carbon. Table 3 summarizes the final concentrations of nitrate reached in the solution and the corresponding percentages of removal for all the initial concentrations studied. As can be observed, activated carbon 1 offered a higher adsorption yield than the other type of carbon.

#### 3.2.2. Kinetic Characterization of the Process

**Figure 5:**
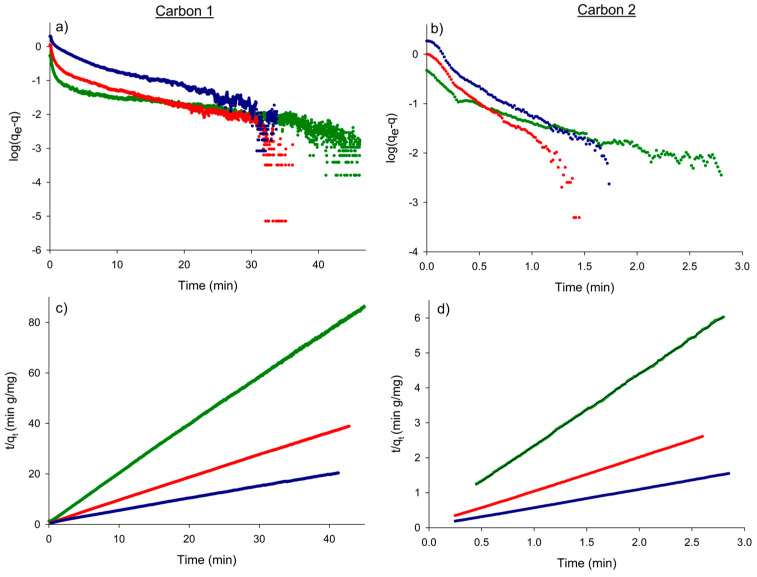
Kinetic plots of the pseudo-first order (**a**,**b**) and pseudo-second (**c**,**d**) order models for nitrate removal by adsorption on activated carbons 1 and 2. Nitrate concentration: 1 × 10^−5^ M (green), 2 × 10^−5^ M (red) and, 4 × 10^−5^ M (blue).

Finally, the experimental concentration–time data were used for further fitting to two of the main kinetic models used for nitrate adsorption: the pseudo-first and the pseudo-second order kinetic models [17,30,31,32,33,34,35]. The following equations have been used for these kinetic models, respectively [36]:

(3) $$\log\left(q_{e}-q_{t}\right)=\log\left(q_{e}\right)-\frac{k_{1}t}{2.303}$$

(4) $$\frac{t}{q_{t}}=\frac{1}{k_{2}q_{e}^{2}}+\frac{t}{q_{e}}$$

where *t* (min) is the time from the start of the nitrate removal process, $q_{t}$ and $q_{e}$ are the amounts of nitrate adsorbed (mg g^−1^) at time “t” and at equilibrium, respectively, and $k_{1}$ (min^−1^) and $k_{2}$ (g mg^−1^ min^−1^) are the pseudo-first order and pseudo-second order rate constants, respectively. The results of the fitting to Equations (3) and (4) are shown in Figure 5 for the different initial concentrations and the two activated carbons used. The data clearly followed the pseudo-second order kinetic model, with excellent linearity over the entire time interval. This is consistent with previously reported nitrate adsorption models [36]. The corresponding kinetic constants $k_{2}$ and the equilibrium parameter $q_{e}$ are given in Table 4. Note that, according to Figure 3, the rate constants for activated carbon 2 were quite higher than those for activated carbon 1. This faster adsorption may be due to the powdery appearance of this adsorbent, as well as the acid pre-treatment carried out by the manufacturer.

### 3.3. Nitrate Removal by Ion Exchange Resin

#### 3.3.1. Potentiometric Response for Exchangeable Chloride and Hydroxide Anions

The utility of the nitrate selective electrode for monitoring the removal of nitrate from aqueous solutions using an anion exchange resin was also evaluated. The ion exchange reaction, unlike adsorption with activated carbon, alters the composition of the solution sample during the process by incorporating into it an amount of ionic species leaving the resin equivalent to the amount of nitrate entering it. This modification of the sample could affect the correct monitoring of nitrate removal, due to the possible contribution of the leaving ions to the potential of the electrode. Therefore, the ability of the nitrate selective electrode to discriminate between the two ions, the nitrate and the ions leaving from the resin was investigated.

The potential of the nitrate selective electrode in the presence of nitrate and the ions leaving from the resin (j) can be described by the Nikolsky–Eisenmann Equation (5):

(5) $$E=E^{0\prime }+S\;log(C_{nitrate}+K_{nitrate,j}\;C_{j})$$

where $K_{nitrate,j}$ is the selectivity coefficient for j of the nitrate electrode. The values of $K_{nitrate,j}$ were determined using the separate solution method, with the potential values measured from chloride at a concentration of 5 × 10^−^^3^ M and hydroxide alone. The nitrate equivalent concentrations corresponding to these potential values were calculated from the nitrate calibration curve and the corresponding selectivity coefficients were then obtained directly from the following relationship (6):

(6) $$K_{nitrate,j}=\frac{C_{nitrate,eq}}{5\times 10^{-3}}\;.$$

The values obtained were 0.01 for chloride and 0.003 for hydroxide. This means that the presence of chloride or hydroxide together with nitrate at the same concentration would cause an error of 1% or 0.3%, respectively, in the determination of nitrate.

As a result of the ion exchange taking place during the nitrate removal process, the concentrations of chloride or hydroxide ions in the solution increase over time as the concentration of nitrate decreases. Therefore, the potential interference with potentiometric monitoring of nitrate removal would increase with time. Thanks to this, the maximum value of the nitrate concentration ratio *n* ($n=C_{nitrate}^{initial}/C_{nitrate}^{final}$) that can be monitored without exceeding a predetermined error can be calculated.

The molar concentration of Cl^−^ or OH^−^ ions in the aqueous solution during the removal process must be equal to the concentration of nitrate removed.

(7) $$C_{j}=C_{nitrate}^{initial}-\frac{C_{nitrate}^{initial}}{n}$$

With a 5% error in the determined nitrate concentration, the following expressions can be written:

(8) $$K_{nitrate,j}\;C_{j}=0.05\;C_{nitrate}^{final}$$

(9) $$K_{nitrate,j}\left(C_{nitrate}^{initial}-\frac{C_{nitrate}^{initial}}{n}\right)=0.05\frac{C_{nitrate}^{initial}}{n}$$

(10) $$n=\frac{0.05+K_{nitrate,j}}{K_{nitrate,j}}.$$

The calculated values of n were 6 and 18 for chloride and hydroxide, respectively. This means that the final nitrate concentration can be up to 6 and 18 times lower, respectively, than the initial nitrate concentration value without interfering with nitrate potentiometric monitoring when the resin is in the form of chloride or hydroxide.

#### 3.3.2. Potentiometric Monitoring of Nitrate Removal

**Figure 6:**
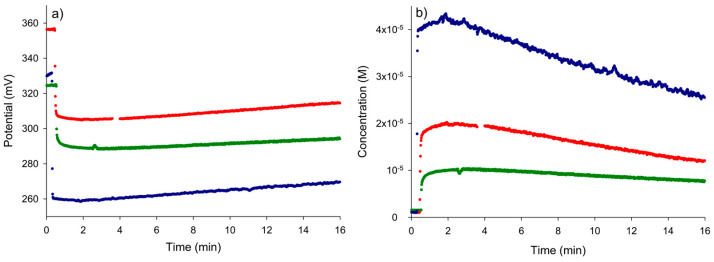
(**a**) The transient potential response during the process of removal of nitrate with the anion exchange resin. (**b**) A concentration–time graph resulting from Figure 6a. Nitrate concentration: 1 × 10^−5^ M (green), 2 × 10^−5^ M (red) and 4 × 10^−5^ M (blue).

The potential versus time and the corresponding nitrate concentration versus time curves for the removal of nitrate by ion exchange resin in a hydroxide form are shown in Figure 5a and Figure 5b, respectively. As in Section 3.2.1, the injection of nitrate into pure water caused an initial rapid decrease and stabilization of the potential. Then, the addition of the resin caused a continuous increase in the potential, corresponding to a continuous decrease in the concentration due to the ion exchange process. The effectiveness of the nitrate removal process over the monitored period was slightly lower for the lowest initial concentration of nitrate studied (25%) than for the other two concentrations (40%) (see Table 5). However, it should be noted that the percentages of removal with the resin could be higher for longer contact times with the sample, since the steady-state potentials were not fully reached for the conditions shown in Figure 6. Nevertheless, the duration of the testing process (16 min) was sufficient to confirm the potential use of the resin for nitrate removal, with the final concentrations of nitrate in the solution still high enough (*n* < 1) to ensure non-interference of the foreign ion OH- with the measurement of the potential.

#### 3.3.3. Kinetic Characterization of the Process

**Figure 7:**
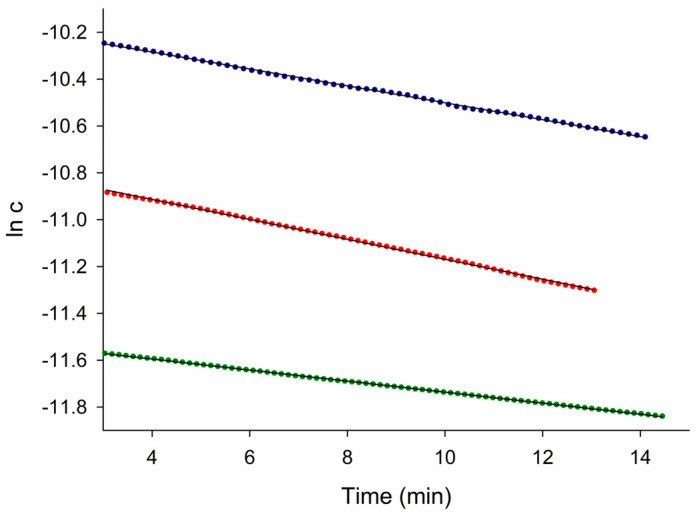
Kinetic plots of the pseudo-first order model for nitrate removal by ion exchange with the resin. Nitrate concentration: 1 × 10^−5^ M (green), 2 × 10^−5^ M (red) and 4 × 10^−5^ M (blue).

After smoothing the raw concentration–time data (shown in Figure 6b), the logarithms of the concentration of nitrate during the removal process with the resin were fitted to the following expression for the pseudo-first order model:

(11) $$\ln C_{nitrate}=\ln C_{nitrate}^{initial}-kt$$

where k is the kinetic constant of the ion exchange process. As shown in Figure 7, the goodness of the fit corroborated the pseudo-first order model. The resulting kinetic constants are gathered in Table 6.

### 3.4. Potential Applications for Nitrate Removal in Real Water Samples

Although the application of the proposed method to the monitoring of nitrate removal in real water samples is beyond the scope of the present work, a discussion of its potential application is given below.

In general, the applicability of the method to real water will depend on the chemical composition of each particular water sample. This covers a wide range of situations, including drinking water, irrigation water, sea water and polluted water. In the case of drinking water [37] and irrigation water [38], the main anions that may accompany nitrate are hydrogen carbonate, sulphate and chloride. The direct application of the method presented in this paper will depend on the concentration ratios between these anions and nitrate. Solids in suspension would not be a problem, as we discussed above. Sulphate should also not be a problem as it interferes very little with nitrate selective electrodes based on quaternary ammonium salts and NPOE as plasticizer [38]. In the case of water samples with high hydrogen carbonate and/or chloride/nitrate ratios, biased results could be obtained. However, the interference of hydrogen carbonate could be eliminated by acidifying the samples with acetic acid. In addition, the interference of chloride could be eliminated by chloride precipitation with silver sulphate. Regarding the presence of organic compounds in polluted water samples, they should not cause interference if they are in neutral or cationic form. On the other hand, some anionic organic compounds such as anionic surfactants may interfere to some extent.

## 4. Conclusions

The constructed nitrate selective electrode allows in situ real-time potentiometric monitoring of nitrate removal from aqueous solutions in batch mode with activated carbon and with anion exchange resin in its hydroxide form. The obtained results show pseudo-second order adsorption kinetics for the activated carbons and pseudo-first order ion exchange kinetics for the resin. The methodology used has a great advantage over other methodologies used for monitoring nitrate removal, in that it is carried out continuously in the same reaction medium and it does not require prior separation of the adsorbent material. We believe that this methodology can be applied to other ions with their corresponding selective electrode and to other adsorbent materials.

## Figures and Tables

**Table 1:** The fitting parameters and coefficients of determination for the calibration plots over the wide concentration range, separated by six weeks.

Calibration	$E^{0´}$ (mV)	*S* (mV/dec)	*R* ^2^
1	−2.5	−57.0	1.0000
2	1.5	−57.9	1.0000

**Table 2:** The fitting parameters and coefficients of determination for the calibration plots over the narrow concentration range for two different membranes.

Calibration	$E^{0´}$(mV)	*S* (mV/dec)	*LD* (M)	*R* ^2^
1	95.1	−44.0	3.5 × 10^−7^	0.9998
2	35.7	−49.0	4.4 × 10^−7^	0.9990

**Table 3:** The final concentrations and percentages of removed nitrate for both types of activated carbon and the different initial concentrations of nitrate assayed. The values between parentheses correspond to the red curves in Figure 4.

*C*_initial_ (M)	Carbon 1		Carbon 2	
	*C*_final_ (M)	Removed Nitrate (%)	*C*_final_ (M)	Removed Nitrate (%)
1 × 10^−5^	1.6 × 10^−6^	84	3.3 × 10^−6^	67
2 × 10^−5^	2.2 × 10^−6^ (2.7 × 10^−6^)	89 (87)	3.8 × 10^−6^ (3.6 × 10^−6^)	81 (82)
4 × 10^−5^	7.3 × 10^−6^	82	1.1 × 10^−5^	74

**Table 4:** The values of the kinetic constants ($k_{2}$), the amount of nitrate adsorbed at equilibrium ($q_{e}$) and the correlation coefficients (*R*^2^) resulting from the fitting of the nitrate removal by adsorption on activated carbons to the pseudo-second order kinetic model. The values of $k_{2}$ and $q_{e}$ indicated between parentheses correspond to the red curves in Figure 4.

*C*_initial_ (M)	*k*_2_ Carbon 1 (g mg^−1^ min^−1^)	*k*_2_ Carbon 2 (g mg^−1^ min^−1^)	*q_e_* Carbon 1 (mg g^−1^)	*q_e_* Carbon 2 (mg g^−1^)	*R*^2^ Carbon 1	*R*^2^ Carbon 2
1 × 10^−5^	2.7	14	0.53	0.49	0.9997	0.9998
2 × 10^−5^	1.3 (1.1)	12 (9.9)	1.1 (1.1)	1.0 (1.0)	0.9999	0.9999
4 × 10^−5^	0.31	6.2	2.1	1.9	0.9998	0.9999

**Table 5:** The final concentrations and percentages of nitrate removed with the anion exchange resin for the different initial concentrations of nitrate assayed.

*C*_initial_ (M)	*C*_final_ (M)	Removed Nitrate (%)
1 × 10^−5^	7.5 × 10^−6^	25
2 × 10^−5^	1.2 × 10^−5^	40
4 × 10^−5^	2.4 × 10^−5^	40

**Table 6:** The kinetic constants and correlation coefficients of the fitting of the concentration–time data for the removal of nitrate with resin to the pseudo-first order model (Equation (11)).

*C*_initial_ (M)	*k* (min^−1^)	*R* ^2^
1 × 10^−5^	0.024	0.9984
2 × 10^−5^	0.043	0.9991
4 × 10^−5^	0.036	0.9995

## Data Availability

The original contributions presented in the study are included in the article, further inquiries can be directed to the corresponding author.
